# Emergency incisional hernia repair: a difficult problem waiting for a solution

**DOI:** 10.1186/1750-1164-6-1

**Published:** 2012-01-04

**Authors:** Hasnain Zafar, Masooma Zaidi, Irfan Qadir, Ayaz Ahmed Memon

**Affiliations:** 1 General Surgery Department, Aga Khan University Hospital, Stadium Road Karachi, Pakistan

**Keywords:** Obstructed hernia, Incisional hernia, Emergency hernia repair

## Abstract

**Background:**

Emergency repair of incarcerated incisional hernia with associated bowel obstruction in potentially or contaminated field is technically challenging due to edematous, inflamed and friable tissues with occasional need for concurrent bowel resection and carries high rates of post-operative infectious complications. The aim of this study was to retrospectively assess the wound related morbidity of use of permanent prosthetic mesh in emergency repair of incarcerated incisional hernia with associated bowel obstruction. We also describe a new technique of leaving the mesh exposed to heal by secondary intention with granulation tissue.

**Methods:**

Between 2000 and 2010 a total of 60 patients underwent emergency surgery for incarcerated incisional hernia with associated bowel obstruction with placement of permanent prosthetic mesh. The wound was closed after hernia repair in 55 patients while it was left open to granulate in 5 patients.

**Results:**

In the group of patients with primary wound closure, 11 patients developed superficial surgical site infection, 5 developed deep wound infection and one patient had cellulitis. These patients were treated with wound debridement and antibiotics. Mesh removal was required in one patient. There were no infections in the group of patients who had their surgical wounds left open. One patient in this group died on the fifth postoperative day from septicemia.

**Conclusion:**

Use of permanent prosthetic mesh in emergency repair of incarcerated incisional hernia with associated bowel obstruction. in contaminated field is associated with high risk of wound infection.

## Background

Incisional hernias occur as a complication in up to 20% of abdominal surgeries [1]. Emergency repair of incarcerated incisional hernia with associated bowel obstruction. in contaminated field is technically challenging due to edematous, inflamed and friable tissues with occasional need for concurrent bowel resection and therefore high rates of post-operative infectious complications [2].

A vast amount of literature advocates the use of prosthetic mesh in clean operative fields; in contrast utilization of prosthetic grafts in contaminated/obstructed settings has been seldom described [3]. Most surgeons believe that permanent prosthetic materials for incisional hernia repair are contraindicated in the setting of gross contamination, which includes emergency presentation of incarcerated incisional hernia with associated bowel obstruction as a subset, due to risk of infection as high as 10% to 35% [4]. However, primary repair of incisional hernia has high recurrence rates, ranging between 10% and 50%; while adoption of prosthesis lowers recurrence rates to between 3% and 18% [5-7]but also increases the risk of local complications such as wound infections. Inspite of possible wound complications, the use of mesh in emergency repair of incarcerated incisional hernia with associated bowel obstruction is sometimes necessitated by the size or nature of defects and often the choice is between absorbable versus non-absorbable mesh in obstructed settings. There is little published literature evaluating various surgical approaches to these incredibly challenging patients. Therefore this is a subject of debate whether to use non-absorbable prostheses with obstructed or gangrenous bowel in potentially or truly infected operating fields [3].

With this background, we undertook this study to focus on a subset of patients who present with incarcerated incisional hernia with associated bowel obstruction and require emergency surgery. We reviewed our institution's experience with the use of permanent prosthetic mesh in such patients, taking into account the wound related complications as the primary outcome. We also describe a new technique of leaving the wound open to allow granulation tissue to grow over the mesh and heal secondarily.

## Methods

We retrospectively reviewed the medical records of all the patients who underwent emergency surgery for incarcerated incisional hernia with associated bowel obstruction with placement of permanent prosthetic mesh in our institution between 2000 and 2010. Pertinent details recorded were baseline demographics, abdominal surgical history, reason for contamination, operative technique and details, postoperative mesh-related morbidity in terms of wound related outcome and mortality. Postoperative follow-up was achieved by review of notes of patient's clinic visits at our hospital since the surgery.

We divided our patients into two groups on basis of wound closure. In one group, a 90 gram prolene mesh was fixed over the anterior rectus sheath after primary repair of hernia (standard onlay technique)and the wound was closed in layers. In other group, mesh was placed over the primarily repaired defect by onlay technique but the wound was left open. The wound was treated by daily dressings until complete neoepithilization of the wound i.e healing by secondary intention. All patients received prophylactic antibiotics, a triple regime consisting of kefzole, augmentin and ceftriaxone. In patients with strangulation who required bowel resection, antiobiotics were continued for five days.

The data was analyzed in SPSS version 17. Patient demographics were presented as percentages for discrete variables and mean (± SD) for continuous variables.

## Results

A total of 60 patients were identified who underwent emergency surgery for incarcerated incisional hernia with associated bowel obstruction with placement of permanent prosthetic mesh. The wound was closed after hernia repair in 55 patients while it was left open to granulate in 5 patients. Table 1 shows the patient characteristics in two groups.

**Table 1:** Patient related factors in the two groups

Factors	Groups	Wound closed		Wound left open	
		
		Bowel resection n = 13	No bowel resection n = 42	Bowel resection n = 2	No bowel resection n = 3
Age	Mean(years ± SD)	52.4 ± 15	53.4 ± 12.5	59 ± 5.7	54.3 ± 9
Gender	Male	3	5	0	1
	Female	10	37	2	2
BMI	< 30	6	16	0	2
	> 30	7	26	2	1
DM	Yes	3	11	1	0
Recurrent Hernia	Yes	5	9	1	1
Hospital Stay	Mean (days ± SD)	5.8 ± 2.8	4 ± 2.1	7 ± 2.8	4.7 ± 2.1
Wound infection	Yes	5	12	0	0
Recurrence	Yes	0	4	0	0

The mean operative time was 170 minutes (SD ± 37). 17 (28.3%) patients had small size hernias (less than 5 cm). Of these 7 patients had single defect and 10 had multiple defects. 12 (20%) patients had medium size hernias (5-10 cm). Of these 7 patients had single defect and 5 had multiple defects. 5 (8.3%) patients had large hernias (more than 10 cm). 3 patients had single and 2 had multiple defects whereas missing data was encountered in 26 cases.

The mean length of hospital stay for all patients was 4.5 days (SD ± 2.4 days). The group of patients who had their wounds left open stayed longer (mean 5.6 ± 2.4 days) compared to the group of patients in whom the wound was closed primarily (mean 4.4 ± 2.3 days). However, the difference in the length of hospital stay between the two groups was insignificant (p value 0.868). During the subsequent clinic visits after discharge from the hospital, wound care was provided. The average duration of follow-up was 3.8 months (range 1-48 months).

Wound infection was defined as any wound that required the prescription of antibiotics and/or skin opening with or without debridement. There were no infections in the group of patients who had their surgical wounds left open. In the other group of patients with primary skin closure, seventeen out of 55 patients (31%) developed surgical wound complications. This group was further subdivided on the basis of need for concurrent bowel resection. In this group, 13 patients required concurrent bowel resection while 42 did not. In the subgroup of patients without concurrent bowel resection 12 developed wound complications (28.5%). Of these, 8 patients developed superficial surgical site infection which required wound debridement. Three of the four patients who developed deep infection were treated with local wound-care measures but one patient required complete removal of mesh. In the other subgroup of patients with concurrent bowel resection, two of the three patients developing superficial surgical site infection required wound debridement. One patient had deep infection and cellulitis each but none required surgical treatment.

One patient in the open wound group died on the fifth postoperative day from septicemia. This 63 year old female presented with strangulated incisional hernia and underwent emergency surgery for bowel resection with stoma formation and mesh placement for abdominal wall defect.

## Discussion

The principles of incisional hernia repair in the setting of surgical field contamination involve the removal of the source of contamination and the reconstruction of the abdominal wall. These operations are challenging and often result in complications that lead to both surgeon and patient frustration [1].

Colonic operations are classified as contaminated and infected (class 3-4) procedures according to Altemeier classification. For this reason the use of mesh in potentially contaminated procedures has been strongly discouraged [7]. Morris et al. [8] suggest abandonment of the use of mesh for repairs in which open bowel is encountered. A trend of increased pain and more severe wound infections after mesh repair were the basis for discontinuation of randomized control trial by Korenkov et al., [9] highlighting the risk of using a foreign body in a hernia repair. Temudom et al. [10] in a series of 50 complex prosthetic giant ventral hernia repairs reported that the two patients with simultaneous bowel surgery subsequently required mesh removal. Others [9,10]recommend that intestinal resection be done first and hernia repair should be postponed for a second time.

The use of mesh is sometime necessitated by the size and the nature of defect. More than half of the patients in our study had either large or multi-loculated hernias. The defects were too large to be repaired primarily. McLanahan et al. [11] reported no increased infectious risk with the prosthetic placement in a series of mostly clean-contaminated wounds having mesh incisional herniorrhapy. Vix et al. [4], Birolini et al. [12], Geisler et al. [13] and more recently Machiaras et al. [7] report 10.6%, 20%, 7% and 15.7% wound related morbidity respectively with the use of mesh in clean-contaminated and contaminated procedures. Campanelli et al. [14] performed ten prosthetic hernia repairs in potentially contaminated areas and report that there were no major or minor complications after a 21 months follow-up period. These authors advocated the use of non-absorbable mesh in potentially contaminated and contaminated operations including colonic resections, with results as good as those observed in clean procedures.

The overall 28.3% infection rate in this study is significantly higher compared to the previous studies. Kelly et al. [3] reported 21% infection rate in a series of emergency and elective incisional hernia repairs. Infection rates were 21% and 4% as reported by Alaedeen et al [1] and Ahmed et al. [15] in a similar patient casemix. The higher than usual infection rate in this study is attributable to the unique set of patients. We focused exclusively on a subset of incisional hernia repair cases which presented with an obstructed bowel and required emergency surgery, which carry higher risk of post-operative complications and have less favorable outcome [2]. Davies et al [2] found 10% infection rates in patients requiring emergency repair for all abdominal hernias. None of above quoted studies has focused on the subset of patients addressed in our study. Most of these studies include elective contaminated cases as a bulk of their population.

In view of the high infection rates, various techniques for mesh placement, including onlay, sublay (retromuscular or extrafascial), or underlay (intraperitoneal or subfascial), have been investigated. Rives-Stoppa technique has been advocated to have low infection rates, ranging between 2% and 17% [16]. However, most of these studies did not focus exclusively on emergency repair of incarcerated hernias. In addition, this procedure is time consuming, as shown by longer mean length of operation time (131 minutes primary, 141 minutes mesh, 231 minutes Stoppa) in a study by Veillette et al [17]. Patients with incarcerated incisional hernia with bowel obstruction are usually hemodynamically unstable. Therefore utility of time-consuming Stoppa technique in such patients is yet to be established.

The infection rate also depends on the need for concurrent bowel resection. It is 38% for patients needing concurrent bowel resection and 28% for those without bowel resection. The high wound infection in patients without bowel resection is postulated to be secondary to bacterial translocation thus an obstructed bowel is a significant risk factor for wound infection.

However, in view of the high infection rates, the option in operating room with a large defect and potentially contaminated field is to use absorbable mesh for temporary closure and do a definitive repair as a second planned operation.

In this study, the wound was left open in five patients to heal secondarily with granulation tissue. The decision to do so was made intra-operatively in light of grossly contaminated surgical field which made placement of prosthetic mesh a risky option. In addition absorbable (biological) mesh is not available in Pakistan. Post-operatively, the wounds took 4-5 months to heal depending upon size of the defect. These patients were advised to continue routine activities and once or twice daily self-wound care using a guaze once granulation had occurred. Patients were followed in the clinic on monthly basis till the wound healed completely. We did not have chronic draining sinuses in our patients.

Figure 1

**Figure 1:**
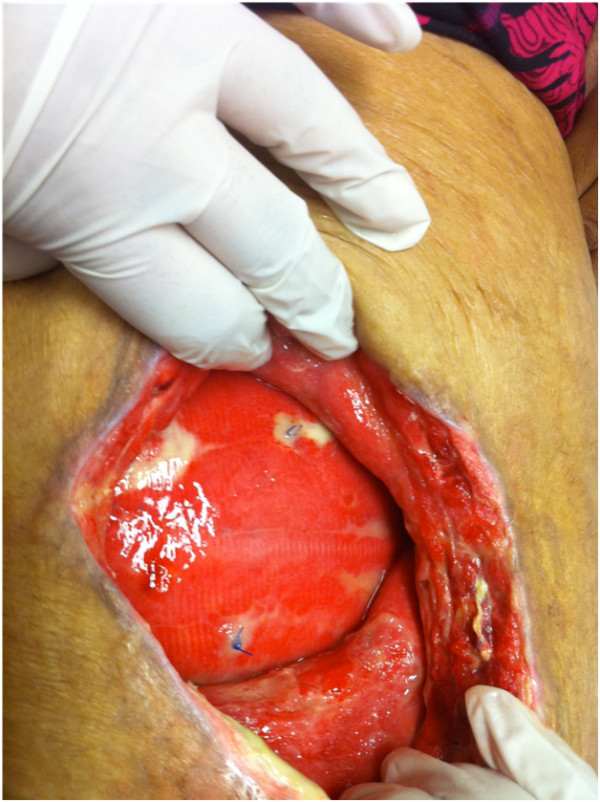
**Healthy pink granulation tissue covering the mesh**.

In our experience, use of prolene mesh in contaminated fields is associated with high rates of wound infection, however mesh removal is rarely needed. All except one patient were treated with wound debridement or antibiotics. The evidence that prolene mesh is resistant to infection is also borne out by studies on Lichenstein hernia repair [18]. The authors in view of the physical characteristics of prolene mesh, that is the mononfilament structure that allows the neutrophils and macrophages to eradicate bacteria, placed the mesh in an onlay position and allowed the wound to granulate and incorporate the mesh. The growth of granulation tissue through the intricese of the mesh is a unique phenomenon. This requires daily change of wound dressing and complete wound healing can take up to a year. The mesh eventually gets incorporated and epithelilized. In one patient mismatch in the wound contraction and mesh contraction resulted in partial auto explanation of the mesh as shown in the picture; this was excised.

**Figure 2:**
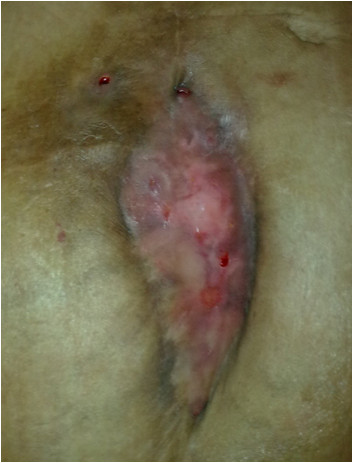
**Mesh partially incorporated with central part auto explanted due to wound contraction**.

**Figure 3:**
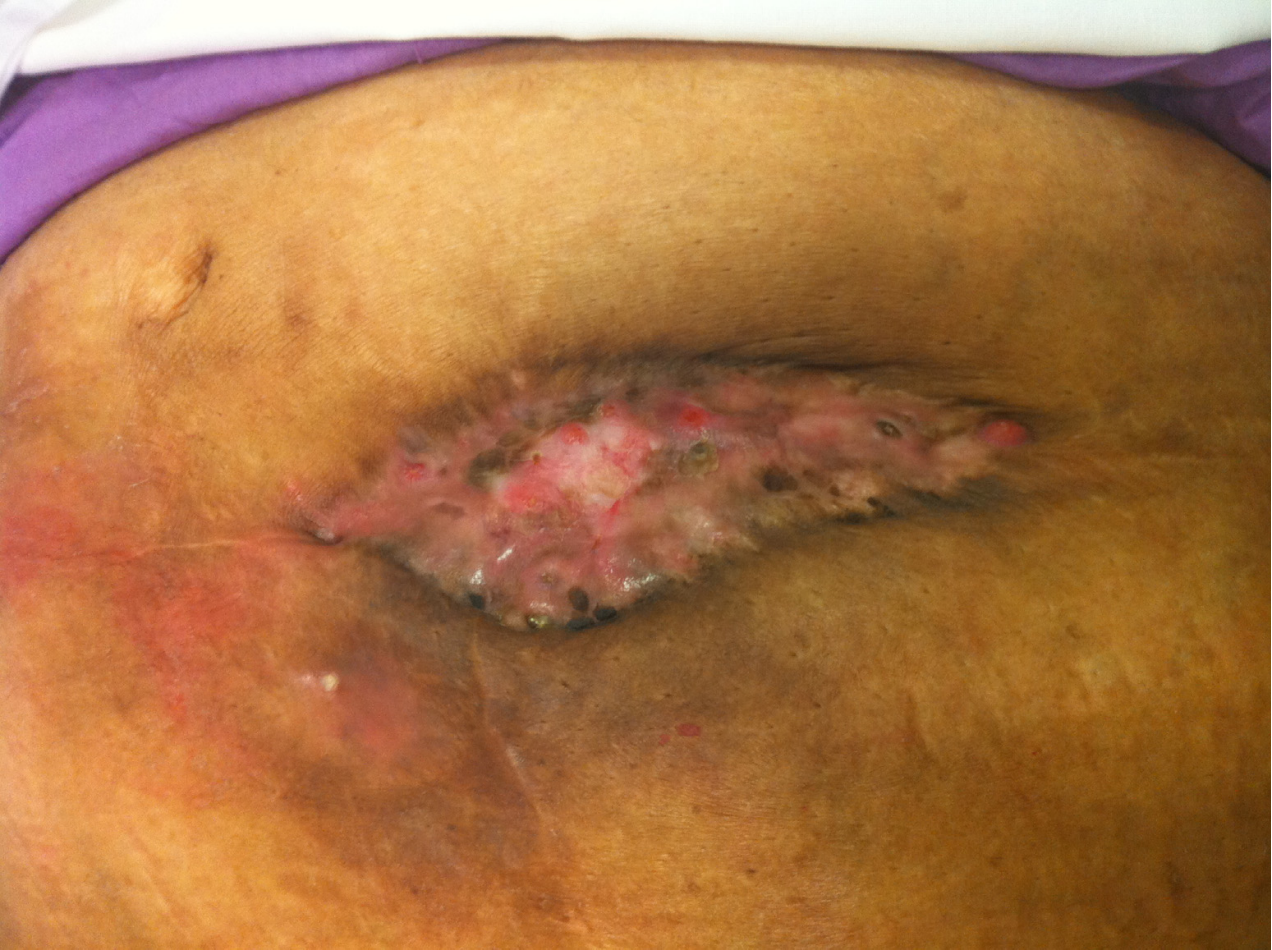
**Final outcome of leaving the mesh exposed showing neo-epithelization in a patient after 5 years**.

Figure 2 and Figure 3

## Conclusion

The retrospective nature and small sample size of this study limits the conclusions that can be drawn regarding the rationale for which procedure should be applied to each individual patient. From our results, we confirm a high rate of wound infection in mesh repair of incarcerated incisional hernia with associated bowel obstruction. The infection rates are even higher in patients undergoing simultaneous bowel surgery. However, mesh explanation is rarely needed even after wound infection. Five patients outcome cannot be used to advocate the use of this new technique of leaving the mesh exposed to heal secondarily. However, this study highlights the need for future prospective studies to evaluate the long term results and reccurence rates of this technique.

### Consent

Written informed consent was obtained from the patients for publication of this report and accompanying images. A copy of the written consent is available for review by the Editor-in-Chief of this journal.

## Competing interests

The authors declare that they have no competing interests.

## Authors' contributions

HZ was involved in patient care and conceived the study and revised the manuscript. MZ and IQ were involved in data collection, literature review and manuscript preparation. AAM participated in the design of the study and performed the statistical analysis. All authors read and approved the final manuscript.
